# Bioinformatics Analysis for Constructing a Six-Immune-Related Long Noncoding RNA Signature as a Prognostic Model of Hepatocellular Carcinoma

**DOI:** 10.1155/2022/2093437

**Published:** 2022-07-07

**Authors:** Jue Wang, Zongrui Jin, Guolin Wu, Jilong Wang, Banghao Xu, Hai Zhu, Ya Guo, Zhang Wen

**Affiliations:** Department of Hepatobiliary Surgery, The First Affiliated Hospital of Guangxi Medical University, Nanning, 530021 Guangxi Province, China

## Abstract

The present study was aimed at identifying the potential prognostic biomarkers of the immune-related long noncoding RNA (IRL) signature for patients with hepatocellular carcinoma (HCC). RNA-sequencing data and clinical information about HCC were obtained from The Cancer Genome Atlas. The IRLs were determined with regard to the coexpression of immune-related genes and differentially expressed lncRNAs. The survival IRLs were obtained using the univariate Cox analysis. Subsequently, the prognosis model was constructed via the multivariate Cox analysis. Subsequently, functional annotation was conducted using Gene Set Enrichment Analysis (GSEA) and principal component analysis (PCA). In total, 341 IRLs were identified, and 6 IRLs were found to have a highly significant association with the prognosis of patients with HCC. The immune prognosis model was constructed with these 6 IRLs (AC099850.4, negative regulator of antiviral response, AL031985.3, PRRT3-antisense RNA1, AL365203.2, and long intergenic nonprotein coding RNA 1224) using the multivariate Cox regression analysis. In addition, immune-related prognosis signatures were confirmed as an independent prognostic factor. The association between prognostic signatures and immune infiltration indicated that the 6 lncRNAs could reflect the immune status of the tumor. Collectively, the present study demonstrates that six-lncRNA signatures may be potential biomarkers to predict the prognosis of patients with HCC.

## 1. Introduction

Liver cancer is a common type of malignant tumor, ranking third for all tumor mortality, accounting for >700,000 deaths and 800,000 new cases each year worldwide [1]. HCC accounts for 75-85% of all primary liver cancer types and is the most common primary malignant tumor. Common risk factors for HCC include hepatitis B virus, hepatitis C virus, chronic drinking, and aflatoxin, amongst others [2]. As the early symptoms of HCC are not obvious, the majority of patients are at the middle and late stages of clinical diagnosis and, as a result, lose the opportunity for surgical treatment [3]. Although chemotherapy and interventional therapy can be used, the survival rate of patients remains low [4]. Currently, surgery is the most effective treatment method for early HCC [5]. It has been reported that sorafenib and lenvatinib have an excellent curative effect for advanced HCC [6, 7]. Due to the high mortality of HCC, it is a necessity to identify valuable targets for promoting the diagnosis, treatment, and prognosis of HCC.

Recent research has suggested that the immune microenvironment has a critical effect on the pathogenesis of HCC [8]. As a typical inflammatory tumor type, immune tolerance and immune escape significantly affect the development of HCC [9]. For instance, myeloid-derived suppressor cells promote tumor development by enhancing the expression levels of immunosuppressive factors [10, 11]. Therefore, further studies are required to determine how to identify immune-related factors.

Long noncoding RNAs (lncRNA) are a class of untranslatable RNA molecules, usually >200 nucleotides in length, that do not encode proteins. Gene regulation mediated by lncRNAs can regulate the expression levels of inflammatory genes in innate immune cells [12]. Previous studies have reported that the abnormal expression of lncRNA is actively involved in tumorigenesis and metastasis [13, 14]. In addition, multiple lncRNAs promote the immune escape of tumor cells [15]. For instance, lnc-EGFR stimulates regulatory T cell (Treg) differentiation and promotes the immune escape of HCC. Furthermore, urothelial cancer associated 1 performs a similar function in gastric cancer cells [16]. Nevertheless, the main mechanism of IRLs in the prediction of HCC remains unknown.

In our research, according to TCGA RNA-sequencing (seq) data, IRLs associated with HCC prognosis were identified using the Molecular Signatures Database (MSigDB), and a prognostic model of HCC was established. The current aim was to use the expression profile of lncRNAs to identify IRLs, which could help to identify a potential prognostic indicator of HCC and a promising target for immunotherapy.

## 2. Material and Methods

### 2.1. Dataset Source and Sample Collection

RNA-seq data for 371 patients with HCC were obtained from TCGA (https://portal.gdc.cancer.gov/) [17]. Data collection was needed to meet the following requirements: the pathological type of all patients was HCC in the dataset; the data included a complete lncRNA expression profile; the data included detailed clinical information and overall survival time, such as sex, age, pathological grade, pathological stage, and TNM stage (Table S1). Samples without complete clinical information and an overall survival (OS) of ≤90 days were excluded. RNA-seq data were HTSeq-FPKM type, not requiring further standardization. Genes with averaged expression values close to zero were deleted. If there are multiple identical genes, the average expression value will be taken as the expression level of the gene. In total, six clinical samples of HCC and paracancerous samples were collected from patients (Table S2). The collected tissues were instantly frozen in liquid nitrogen and stored in a -80°C refrigerator. The study has obtained the patients' informed consent and been approved by the ethics committee of the First Affiliated Hospital of Guangxi Medical University. The research was conducted in accordance with the Declaration of Helsinki.

### 2.2. Screening of Differentially Expressed Immune-Related lncRNAs

Immune-associated genes were acquired from the MSigDB v7.0 (immune system process M13664 and immune response M19817, https://www.gsea-msigdb.org/gsea/msigdb/index.jsp) [18, 19]. In addition, the lncRNA expression data were obtained from TCGA RNA-seq data and the GENCODE database (https://www.gencodegenes.org/) [20]. lncRNAs with an average value of >0.2 were included in the differentially expressed lncRNA (DElncRNA) screening. DElncRNAs were obtained via the Wilcoxon test method in the R package of limma (|log2*FC*| > 1 and false discovery rate (FDR) < 0.05, version 3.42.2, https://bioconductor.org/packages/release/bioc/html/limma.html). Then, IRLs were obtained by establishing the coexpression network of immune genes and lncRNAs (|*R*| > 0.5; *P* < 0.001) using the cor.test function [21] in R.

### 2.3. Cox Regression

lncRNAs related to prognosis were identified via the univariate Cox regression, using the survival package of R (*P* < 0.001). The survival package was used to calculate the risk score via stepwise regression multivariate Cox analysis [22, 23]. The formula used was as follows: Risk score=(*β*_lncRNA1_ × expression level of lncRNA_1_)+(*β*_lncRNA2_ × expression level of lncRNA_2_)+···+*β*_lncRNAn_ × expression level of lncRNA_n_). The median risk score divided the 329 samples into low- and high-risk groups. The Kaplan-Meier survival curve of patients in the two groups was plotted using the survival and survminer packages of R. Based on the six-lncRNA signatures and clinicopathological features, the independent prognostic factors of HCC were analyzed via the Cox regression analyses. The receiver operating characteristic (ROC) curve was used to estimate the accuracy of prognosis prediction within 5 years. All displayed *P* values were two-sided.

### 2.4. Bioinformatics Analysis

Functional annotation was performed between different risk groups via GSEA (http://www.broadinstitute.org/gsea/index.jsp) [24]. The immune system process M13664 and the immune response M19817 (C5) gene sets were downloaded from the GSEA website and MSigDB database. The GSEA version 4.0.3 software was used to analyze the data, and the current study set a random combination of 1,000 analyses. Moreover, PCA was used to evaluate the distribution patterns of various risk groups.

### 2.5. Correlation Analysis of Prognostic Model and Immune Cell Infiltration

The connection between the prognosis model and immune invasion was calculated via TIMER [25], which is a powerful tool to study tumor immune cell infiltration. TIMER involves the infiltration level of six types of immune cells (B cells, CD4^+^ T cells, CD8^+^ T cells, macrophages, neutrophils, and dendritic cells). The immune infiltration level for patients with HCC was obtained via the TIMER database. In addition, the relationship between prognosis signatures and immune infiltration was analyzed with R 3.6.0.

### 2.6. Reverse Transcription-Quantitative (RT-q) PCR

According to the reagent instructions, the total RNA was extracted with RNAiso Plus reagent (Takara Bio, Inc.) and then was reverse transcribed into cDNA using PrimeScript™ RT reagent kit with a gDNA Eraser (Takara Bio, Inc.) at 37°C for 15 min and 85°C for 5 sec. The TB Green Premix Ex Taq™ II kit (Takara Bio, Inc.) was used for qPCR in an ABI 7500 Real-Time PCR system (Applied Biosystems; Thermo Fisher Scientific, Inc.). The 20 *μ*l system included 10 *μ*l TB Green Premix Ex Taq II, 0.8 *μ*l each primer, 0.4 *μ*l ROX Reference Dye II, 2 *μ*l cDNA, and 6 *μ*l double distilled water. The thermocycling conditions were as follows: initial denaturation for 30 sec at 95°C, followed by 40 cycles for 5 sec at 95°C, and 34 sec at 60°C. The primer pairs were as follows: AC099850.4 forward (5′-3′), AGGCTGGAGTGGCAGTGTT and reverse (5′-3′), GTGAGACCTAGTTCCCTGTTGT; negative regulator of antiviral response (NRAV) forward (5′-3′), CTCTGTTCCCAGCCCAGTCCA and reverse (5′-3′), TCCCACAGGGTGCCTTCTTTC; AL031985.3 forward (5′-3′), CTGGTTGAGACCCACTGATGA and reverse (5′-3′) CTTGAGCCAAACGAAACCTAA; PRRT3-antisense RNA (AS)1 forward (5′-3′), GCAAAATGGAGATAACAGCAC and reverse (5′-3′), AGCCTGGATGACAGAGTGAGA; AL365203.2 forward (5′-3′) ACACCCACTGATCCAAAGTCT and reverse (5′-3′), TTCAAATAACATCGTCCACCC; long intergenic nonprotein coding RNA 1224 (LINC01224) forward (5′-3′), CATGTGGGCAAAGCAGA and reverse (5′-3′), TGGGGCATCGTGACATA; and *β*-actin forward (5′-3′), CTACCTCATGAAGATCCTCACCGA and reverse (5′-3′), TTCTCCTTAATGTCACGCACGATT.

### 2.7. Statistical Analysis

In the current research, IRLs were identified via Pearson's correlation analysis. The difference in OS between various risk groups was evaluated via the Kaplan-Meier curve analysis and the log-rank test. The independent prognostic factors for HCC were determined via the Cox regression analysis. Differences in clinicopathological features between groups were tested by an unpaired *t*-test or a Kruskal-Wallis test. Differences between HCC and paracancerous tissues in clinical samples were tested by a paired *t*-test. All statistical analysis was performed on R software 3.6.0. A significant statistical difference was indicated via *P* < 0.05.

## 3. Results

### 3.1. Differential Expression of lncRNAs and Immune-Related lncRNAs in HCC

**Figure 1:**
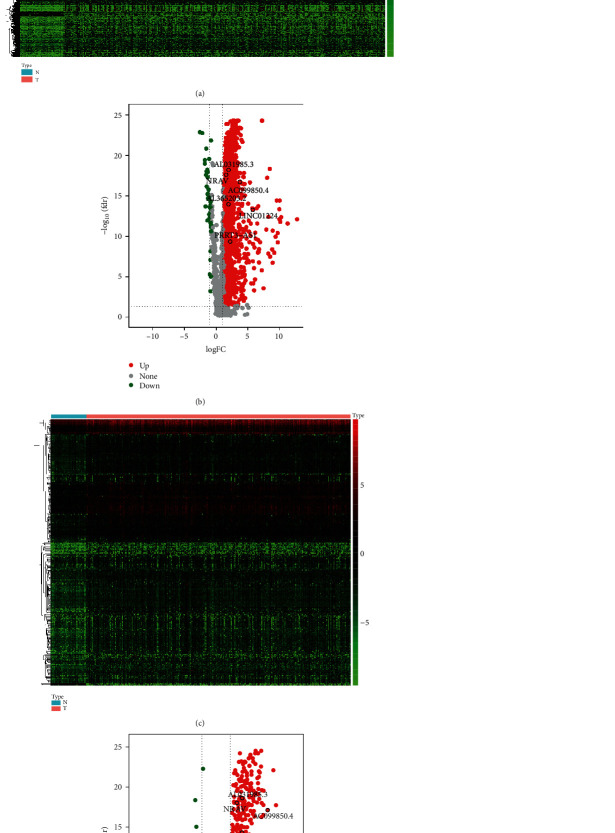
DElncRNAs and immune-related lncRNAs in hepatocellular carcinoma based on The Cancer Genome Atlas dataset. (a) Heatmap of DElncRNAs. The color from green to red displays the trend from low to high expression. (c) Volcano plot of DElncRNAs. Red dots represent upregulated genes and green dots represent downregulated genes. (b) Heatmap and (d) volcano plot of differential expression of immune-related lncRNAs. Six immune-related lncRNAs constructing the prognostic model were upregulated. lncRNA: long noncoding RNA; DElncRNAs: differentially expressed lncRNAs; FC: fold change; FDR: false discovery rate; NRAV: negative regulator of antiviral response; PRRT3-AS1: PRRT3-antisense RNA1; LINC01224: long intergenic nonprotein coding RNA 1224.

Compared with normal samples, 1,479 lncRNAs were screened as the DElncRNAs in HCC samples. The threshold values of screening were |log2*FC*| > 1and FDR < 0.05. Figures 1(a) and 1(b) showed the heatmap and volcano plot of DElncRNAs, respectively. In total, 332 immune genes were screened via the MSigDB v7.0, and IRLs were collected by constructing an immune gene-lncRNA coexpression network. Subsequently, 341 IRLs were identified. The heatmap and volcano plot of IRLs for HCC are presented in Figures 1(c) and 1(d).

### 3.2. Construction of Prognostic Prediction Model

Expression profiles of IRLs were merged with survival data in 329 cases. A univariate Cox regression was performed to obtain 17 lncRNAs associated with patient OS. Then, the interactions of these associated lncRNAs with patient OS were analyzed, and a model composed of six lncRNAs was identified as the best prognosis model for predicting patient OS, as determined via the multivariate Cox regression. A total of six lncRNAs, including AC099850.4, NRAV, AL031985.3, PRRT3-AS1, AL365203.2, and LINC01224, were highly expressed in tumor tissues, based on TCGA cohort analysis (Fig. S1). Moreover, four IRLs were considered as independent prognostic risk factors for HCC in the six lncRNAs. Information regarding the six lncRNA signatures is listed in Table 1.

**Figure 2:**
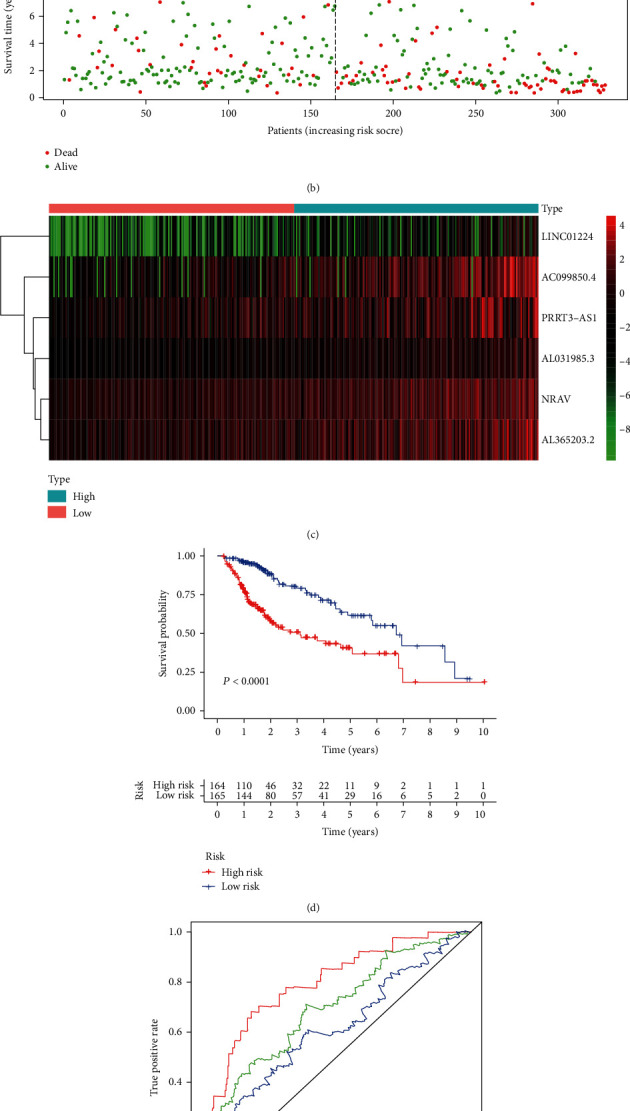
Construction of the prognostic model for HCC in The Cancer Genome Atlas dataset. (a) Distribution of the six-lncRNA risk score. (b) Survival status and overall survival time of patients with HCC. (c) Heatmap of the expression levels of the six lncRNAs. (d) Kaplan-Meier curves for the low- and high-risk groups. (e) ROC analysis for 1-, 3-, and 5-year survival predictions. lncRNA: long noncoding RNA; HCC: hepatocellular carcinoma; NRAV: negative regulator of antiviral response; PRRT3-AS1: PRRT3-antisense RNA1; LINC01224: long intergenic nonprotein coding RNA 1224; ROC: receiver operating characteristic; AUC: area under the curve.

Next, according to the median risk score (Figure 2(a)), 329 liver cancer samples were divided into two groups: the high-risk group (*n* = 164) and the low-risk group (*n* = 165). Figures 2(b) and 2(c) showed the survival profile and gene expression heatmap, respectively. The survival curve suggested that, compared with the low-risk group, the overall survival time of the high-risk group decreased markedly (*P* = 1.54 × 10^−7^; Figure 2(d)). The area under the ROC curve (AUC) was performed to calculate the prediction performance of the six-lncRNA model. The AUC values of the ROC curve for 1-, 3-, and 5-year OS were 0.797, 0.692, and 0.616, respectively, which indicated that the model had good sensitivity and specificity (Figure 2(e)). In addition, it was found that, compared with a single lncRNA, this signature had an improved overall survival prediction ability (Fig. S2).

### 3.3. Independence of Risk Score and Clinical Characteristics

Clinical data of 214 patients with HCC, including sex, age, histological grade, pathological stage, and TNM stage, were collected for the following research. The independent predictive capability of the six-lncRNA characteristics was evaluated via the Cox analysis. Univariate analysis identified that pathological stage, T stage, and the six-lncRNA prognosis model were significantly associated with the OS rate (*P* < 0.001). After the multivariate Cox regression analysis, only the prognosis model of IRLs remained an independent prognostic factor in association with OS (*P* < 0.001; Table 2).

In addition, the current study analyzed the correlation between the prognosis model and clinical features. Our research demonstrated that the risk score was increased remarkably in relation to the following clinical features: female, advanced T stage, and pathological stage (Fig. S3A–C), which further confirmed the clinical application value of the model. It was also found that the expression levels of AC099850.4, AL031985.3, and NRAV were gradually increased in the advanced T stage (Fig. S3D). Moreover, all six lncRNAs were highly expressed in more advanced histological grades (Fig. S3E), which provided insights for further detection of tumor biomarkers.

### 3.4. Immune Status Analysis for Different Risk Groups

**Figure 3:**
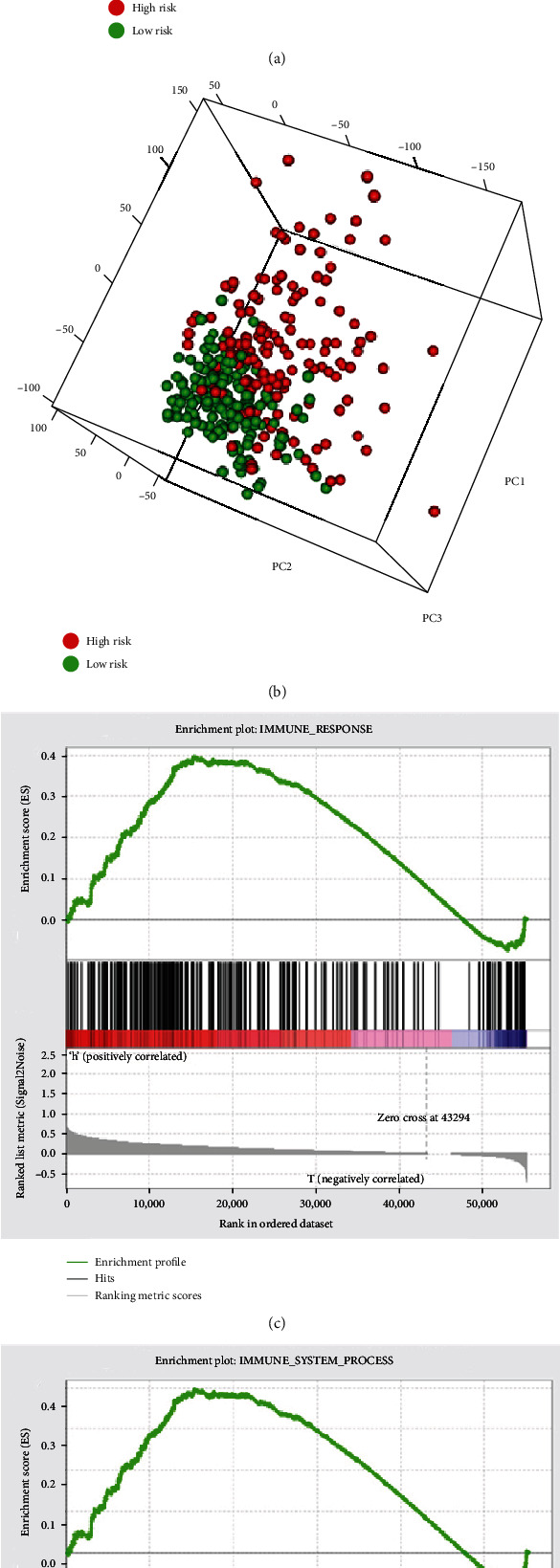
Different immune statuses of the low- and high-risk groups in The Cancer Genome Atlas dataset. PCA analysis between the two groups based on (a) immune genes and (b) all genes. (c and d) Gene Set Enrichment Analysis identified a prominent enrichment of immune-associated phenotypes in the high-risk group. The normalized enrichment scores were 1.28 and 1.47, respectively. PC: principal component.

In accordance with the expression profiles of whole genome and immune-associated genes, different expression patterns were identified between different risk groups via PCA. The results demonstrated that, according to the immune gene sets, the two groups could be distinguished and displayed different immune statuses (Figure 3(a)). However, according to the whole gene expression profile, PCA indicated that there was no significant separation in immune status between these groups (Figure 3(b)). GSEA further verified the functional annotation. In the high-risk group, the immune-associated process and response were more positive (Figures 3(c) and 3(d)). The results suggested that the high-risk group had more immune-related reactions.

**Figure 4:**
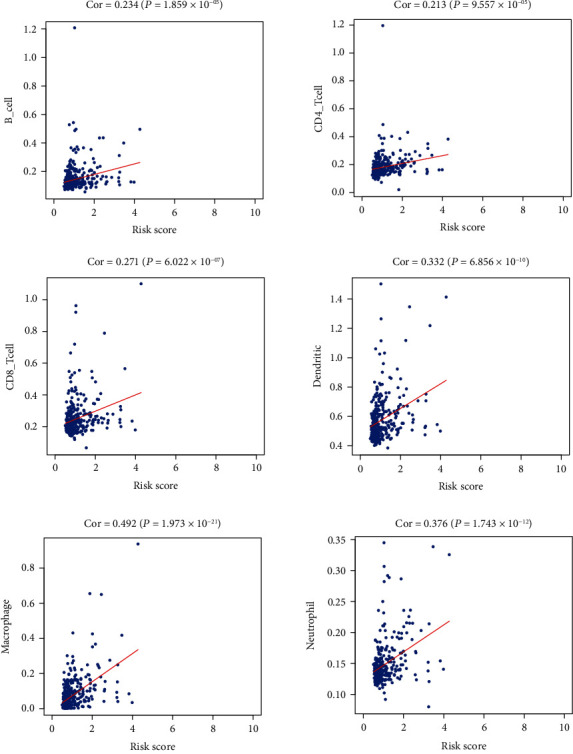
Correlation between the immune-related long noncoding RNA signatures and infiltration abundances of immune cells in The Cancer Genome Atlas dataset. Cor: correlation coefficient.

In order to clarify the momentousness of the IRLs in the tumor microenvironment, the correlation between the six-lncRNA prognostic model and immune infiltration was evaluated. As presented in Figure 4, six types of immune cells were positively correlated with the risk score. Therefore, immune cells may be more active in the high-risk group according to the prognostic signature.

### 3.5. Clinical Validation of the Six lncRNA Expressions

**Figure 5:**
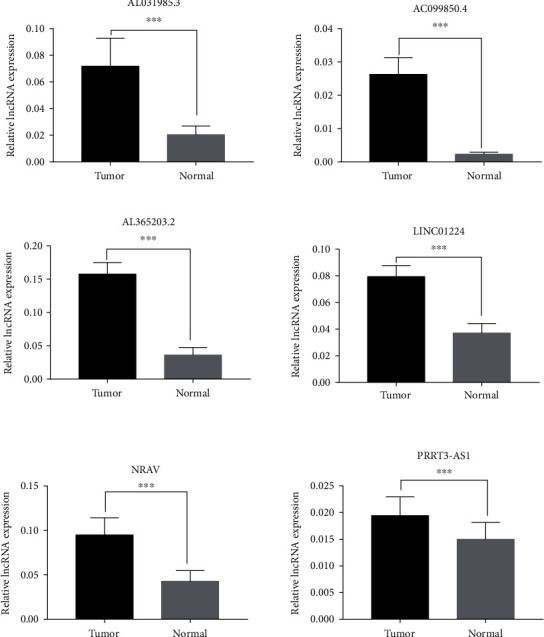
Verification of lncRNA expression levels of the six immune prognostic genes. The lncRNA expression levels of AC099850.4, NRAV, AL031985.3, PRRT3-as1, AL365203.2, and LINC01224 in six pairs of hepatocellular carcinoma and adjacent normal tissues were measured using reverse transcription-quantitative PCR. Data were analyzed using a paired *t*-test. ^∗∗∗^*P* < 0.001. lncRNA: long noncoding RNA; NRAV: negative regulator of antiviral response; PRRT3-AS1: PRRT3-antisense RNA1; LINC01224: long intergenic nonprotein coding RNA 1224.

Overall, six pairs of hepatocellular carcinoma and paracancerous samples were examined to verify the lncRNA expression levels of six genes. The results identified that all six lncRNAs were highly expressed in tumor tissues (*P* < 0.001) (Figure 5). This was in keeping with the current data analysis.

## 4. Discussion

TCGA is a comprehensive database containing multiple cancer expression datasets, and the TCGA lncRNA dataset has been widely used in the diagnosis and prognostic prediction of HCC. The prognosis of HCC remains very poor. At present, treatment of liver cancer mainly includes hepatectomy, liver transplantation, radiofrequency ablation, transarterial chemoembolization, and drug treatment. However, these treatments have limited effect, and so far, there is no effective method for accurately predicting the prognostic signatures of patients with liver cancer. Therefore, it is important to determine reliable biomarkers and identify prediction factors for the OS time for patients with HCC.

Accumulating research has indicated that the immune microenvironment has a great effect on the occurrence, progression, response to treatment, and long-term prognosis of patients with liver cancer [26, 27]. Immune cells, as the monitoring cells of the body, can interfere with molecular signals and recognize the abnormal proliferation of tumor cells. These cells have significant roles in the biological function of cancer, containing tumor proliferation, metastasis, and invasion [28]. Moreover, the presence of the immune escape mechanism often affects the prognosis of the tumor. Multiple immunosuppressive cells of the HCC tumor microenvironment participate in the immune escape of tumor cells [29]. Previous research has suggested that the M2 phenotype in TAM produces an inflammatory environment for tumor growth and promotes tumor progression and metastasis by inducing tumor angiogenesis in HCC, which greatly reduces the survival rate of the patients [30]. However, some cancer cells can avoid being detected by the immune system. These can evade the immune system by inhibiting the immune response, such as antigen presentation, thereby promoting tumor invasion [31]. Recent studies have reported that activation of *β*-catenin promotes immune escape and resistance to programmed cell death 1 (PD-1) and may represent a novel biomarker of T cell rejection in patients with HCC [32]. PD-1, as an immune checkpoint molecule, leads to poor prognosis in patients with cancer by reducing T cell activity and enhancing the immune tolerance of tumor cells. It has also been reported that nivolumab, as an immunosuppressant of PD-1, may play a role in advanced liver cancer [33]. Another study showed that knockout of lncRNA-MM2P could stop the phosphorylation of STAT6, thus preventing the M2 polarization of macrophages driven by cytokines and weakening the tumor angiogenesis function of M2 macrophages [34].

Previous researchers have systematically and comprehensively studied the function of lncRNAs in HCC. For example, it has been shown that the expression levels of lncRNAs may be dysregulated in HCC, which is closely associated with the occurrence, metastasis, therapeutic target, prognosis, or diagnosis of HCC [13, 35]. Previous studies have reported that the highly expressed lncRNAs HULC [36] and HEIH [37] can promote the development of liver cancer. Accumulating evidence has also suggested IRLs are a potential target for tumor therapy, which has predictive value for prognosis. For instance, a variety of lncRNAs, including lncRNA-D16366, lncRNA-ELMO1-AS1, and lncRNA-AWPPH [38], have been considered as potential therapeutic targets and prognostic signatures for liver cancer. Based on these research results, the present study established a new lncRNA prognosis model. As immune-related lncRNAs serve an important role in HCC, it is necessary to identify its potential lncRNA biomarkers. Mining of lncRNA datasets in TCGA-LIHC is a reliable method.

In the present study, 341 immune-associated lncRNAs were obtained via coexpression analysis in the TCGA dataset. In the Cox regression analysis of IRLs, 17 lncRNAs were significantly correlated with OS. Then, six immune-related lncRNAs (AC099850.4, NRAV, AL031985.3, PRRT3-as1, AL365203.2, and LINC01224) were identified as prognostic risk factors for HCC. Based on these six IRLs, 329 samples were divided into high- and low-risk groups by calculating the risk values of all samples. The survival analysis suggested that the prognosis of the high-risk group was worse (*P* = 1.54 × 10^−7^). In addition, all six lncRNAs were risk-related genes (hazard ratio > 1), and these lncRNAs were upregulated in the high-risk group. Using multivariate regression analysis, six immune-related lncRNA signatures were identified as independent prognostic factors for HCC. The survival analysis and AUC value of the ROC curve identified that the six prognosis-associated lncRNA model were credible in predicting the overall survival.

In the current study, PCA and GSEA analyses confirmed that high- and low-risk groups were divided into different immune patterns, and high-risk groups were more active with regard to the immune response and process. This result prompts the further investigation of the potential biological mechanism and clinical significance of the prognosis model in future studies.

In order to understand the characteristics of tumor immunity, the present study examined the interaction between the new prognosis model and immune infiltration. The results suggested that the infiltration degree of six types of immune cells may be higher in high-risk patients. Moreover, all six types of immune cells were positively correlated with the immune associated lncRNA signatures, suggesting that the signatures may be a prediction factor of incremental immune cell infiltration in the tumor microenvironment. This may provide a novel idea for the immunotherapy of liver cancer.

Current studies have shown that DC infiltration is closely associated with the decline of OS in patients with HCC [39], which is in line with the current results. Previous research has shown that neutrophils promote the growth, development, and resistance to sorafenib of HCC by recruiting macrophages and Treg cells [40]. Previous studies have reported that macrophages promote tumor growth and invasion in HCC and lead to a poor prognosis for liver cancer [41]. At present, it has been revealed that the invasion of B cells in HCC can increase tumor invasiveness and reduce disease-free survival [42]. However, the mechanism involving immune cells in liver cancer progression remains unknown. When investigating the mechanism of immune infiltration in tumor progression, it is expected that novel immunotherapeutic targets for HCC will be identified.

In the present study, six immune-associated lncRNA signatures were identified as potential prognostic biomarkers for liver cancer. The current study used six pairs of HCC tissues and paracancerous tissues to evaluate the lncRNA expression levels of the six genes. The results demonstrated that all six lncRNAs were highly expressed in tumor tissues. Moreover, the experimental results were mostly consistent with those of the TCGA database, which confirmed the reliability of the present model. Recent studies have reported that PRRT3-AS1 may be a potential therapeutic target for GBM [43]. Fan et al. [44] revealed that PRRT3-AS1 was highly expressed in prostate cancer (PC). Furthermore, PRRT3-AS1 silencing is able to activate the peroxisome proliferator-activated receptor *γ* gene, which can inhibit PC cell proliferation and promote cell apoptosis and autophagy by blocking the mTOR signaling pathway. In addition, Li et al. [45] reported that LINC01224 may be a potential prognostic marker of breast cancer, while Gong et al. found that LINC01224 was upregulated in HCC. LINC01224 silencing can reduce checkpoint kinase 1 expression by competitive binding to miR-330-5p, thereby inhibiting the progression of HCC [46]. Studies have also shown that the downregulation of NRAV is a part of the host's antiviral defense [47]. However, its role in cancer is yet to be fully clarified.

Based on the current research, the identified lncRNAs may become potential targets for immunotherapy and have great potential for predicting and evaluating the OS of patients with HCC. The advantage of the current study lies in the mining and analysis of TCGA RNA-seq data and the establishment of a novel immune-related prognosis model. This model was powerful in predicting the OS of patients with HCC. Considering the rapid development of immunotherapy for liver cancer in recent years, the present study investigated the relationship between risk score and tumor immune infiltration. However, the disadvantage of the current research was that the model had not been verified by other databases. In the future, the prognostic value of the six-lncRNA signatures will need to be further verified in other independent HCC datasets and larger clinical patients. At the same time, this study lacked the detection of proteomics and immunohistochemistry. Thus, the application value of these immune-related lncRNAs needs to be further clarified in a subsequent study. Despite these limitations, the current research established a novel IRL signature for HCC, which is closely associated with patient risk and OS.

## 5. Conclusion

In conclusion, this study constructed the coexpression network of immune genes and lncRNAs and identified 341 IRLs. Then, we constructed a prognostic model based on the expression of six prognostic lncRNAs. This model could significantly distinguish the high- and low-risk groups of HCC patients, and the prognosis of patients in the high-risk group was worse. Univariate analysis identified that T classification (*P* = 8.94 × 10^−08^), M classification (*P* = 0.009), and the six-lncRNA prognosis model were significantly associated with the OS rate. After the multivariate Cox regression analysis, the prognostic model remained an independent prognostic factor for HCC, which could predict the prognosis better than other traditional clinical indicators. Moreover, the prognostic model was related to the clinical progression of HCC. In addition, the level of immune cell infiltration was higher in the high-risk group. The results would help to establish a reliable risk assessment model and provide new insights into HCC-related immunotherapy strategies.

## Figures and Tables

**Table 1:** Six long noncoding RNA signatures identified by multivariate Cox regression analysis.

Ensemble ID	Gene name	Hazard ratio	*P* value	Coefficient
ENSG00000265415	AC099850.4	1.061603	0.131874	0.060
ENSG00000248008	NRAV	1.106361	0.123935	0.101
ENSG00000260920	AL031985.3	1.395307	0.026389	0.333
ENSG00000230082	PRRT3-AS1	1.097298	0.001096	0.093
ENSG00000273038	AL365203.2	1.089536	0.033453	0.086
ENSG00000269416	LINC01224	1.501971	0.043483	0.407

NRAV: negative regulator of antiviral response; PRRT3-AS1: PRRT3-antisense RNA1; LINC01224: long intergenic nonprotein coding RNA 1224.

**Table 2:** Univariate and multivariate analyses of overall survival in patients with hepatocellular carcinoma from The Cancer Genome Atlas.

Variables	Univariate analysis		Multivariate analysis	
	Hazard ratio (95% CI)	*P* value	Hazard ratio (95% CI)	*P* value
Age (years, increasing years)	0.995 (0.976-1.014)	0.603	1.003 (0.982-1.024)	0.801
Sex (female/male)	0.893 (0.532-1.500)	0.669	1.026 (0.566-1.858)	0.933
Histologic grade (1/2/3/4)	1.083 (0.772-1.520)	0.644	1.160 (0.802-1.678)	0.431
Pathologic stage (1/2/3/4)	2.084 (1.590-2.733)	1.07 × 10^−07^	1.257 (0.432-3.656)	0.674
T classification (1/2/3/4)	1.980 (1.541-2.543)	8.94 × 10^−08^	1.476 (0.555-3.925)	0.435
M classification (0/1)	4.769 (1.485-15.311)	0.009	1.779 (0.454-6.966)	0.408
N classification (0/1)	2.439 (0.593-10.035)	0.217	2.059 (0.314-13.500)	0.452
Prognostic model (low/high)	1.238 (1.163-1.317)	1.69 × 10^−11^	1.189 (1.109-1.275)	1.17 × 10^−06^

T: tumor; N: node; M: metastasis.

## Data Availability

The datasets used and/or analyzed during the current study are available upon reasonable request from the corresponding author.

## Abbreviations

GSEA: Gene Set Enrichment Analysis
lncRNA: Long noncoding RNA
IRLs: Immune-related long noncoding RNAs
OS: Overall survival
PCA: Principal component analysis
HCC: Hepatocellular carcinoma
TCGA: The Cancer Genome Atlas.

## Supplementary Materials

Supplementary Materials Figure S1: differential expression analysis of six long noncoding RNAs in The Cancer Genome Atlas database. Figure S2: survival curves of the six prognostic long noncoding RNAs in hepatocellular carcinoma based on The Cancer Genome Atlas dataset. Figure S3: association between immune-related lncRNAs, risk score, and clinicopathological features in The Cancer Genome Atlas dataset. Table S1: characteristics of the patients with hepatocellular carcinoma in The Cancer Genome Atlas dataset (*n* = 371). Table S2: clinical features of six patients with hepatocellular carcinoma.
