# Supplementary material for: Bioinformatics Analysis for Constructing a Six-Immune-Related Long Noncoding RNA Signature as a Prognostic Model of Hepatocellular Carcinoma
Source: Biomed Res Int. 2022 Jul 7;2022:2093437. doi: 10.1155/2022/2093437 (PMC9283041; doi:10.1155/2022/2093437)
Supplement: Supplementary Materials — Figure S1: differential expression analysis of six long noncoding RNAs in The Cancer Genome Atlas database. Figure S2: survival curves of the six prognostic long noncoding RNAs in hepatocellular carcinoma based on The Cancer Genome Atlas dataset. Figure S3: association between immune-related lncRNAs, risk score, and clinicopathological features in The Cancer Genome Atlas dataset. Table S1: characteristics of the patients with hepatocellular carcinoma in The Cancer Genome Atlas dataset (n = 371). Table S2: clinical features of six patients with hepatocellular carcinoma. [file 2093437.f1.pdf]

Supplementary figures and tables

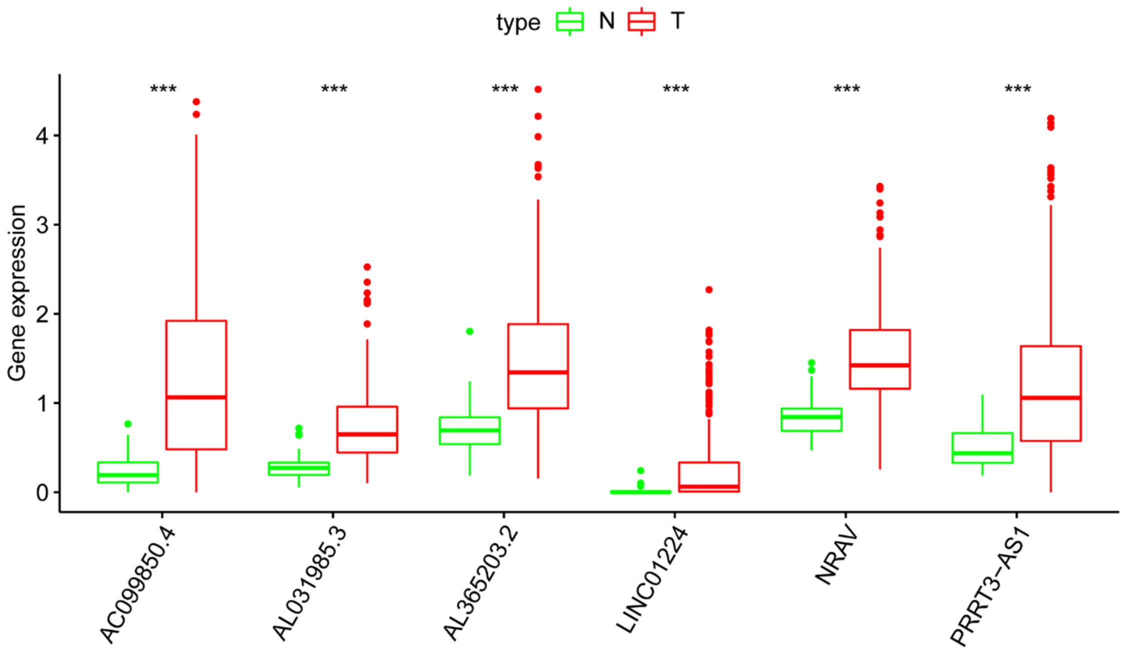

Figure S1. Differential expression analysis of six long non-coding RNAs in The Cancer Genome Atlas database. Data were analyzed using an unpaired t-test. ***P<0.001. NRAV, negative regulator of antiviral response; PRRT3-AS1, PRRT3-antisense RNA1; LINC01224, long intergenic non-protein coding RNA 1224; N, normal; T, tumour.

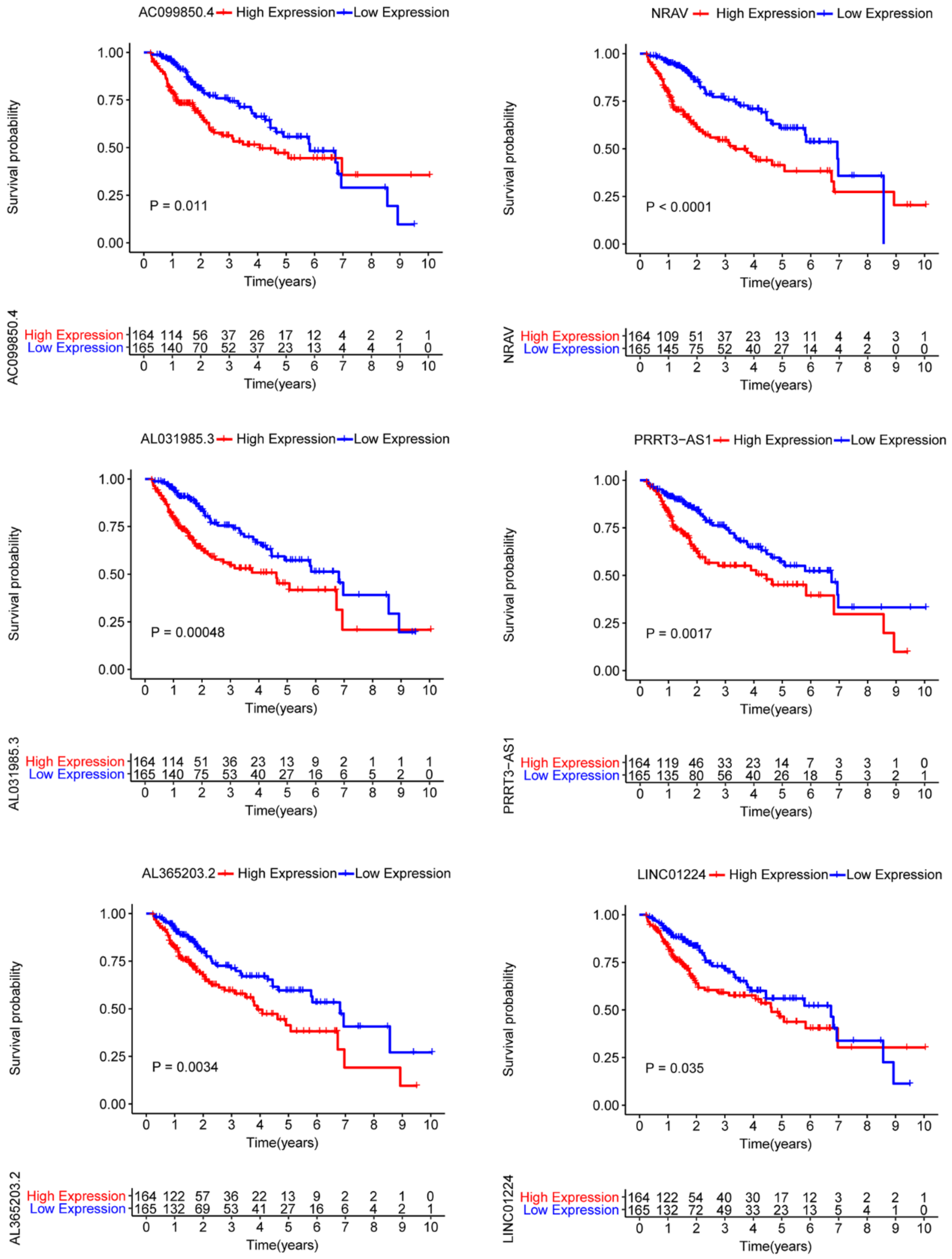

Figure S2. Survival curves of the six prognostic long non-coding RNAs in hepatocellular carcinoma based on The Cancer Genome Atlas dataset. NRAV, negative regulator of antiviral response; PRRT3-AS1, PRRT3-antisense RNA1; LINC01224, long intergenic non-protein coding RNA 1224.

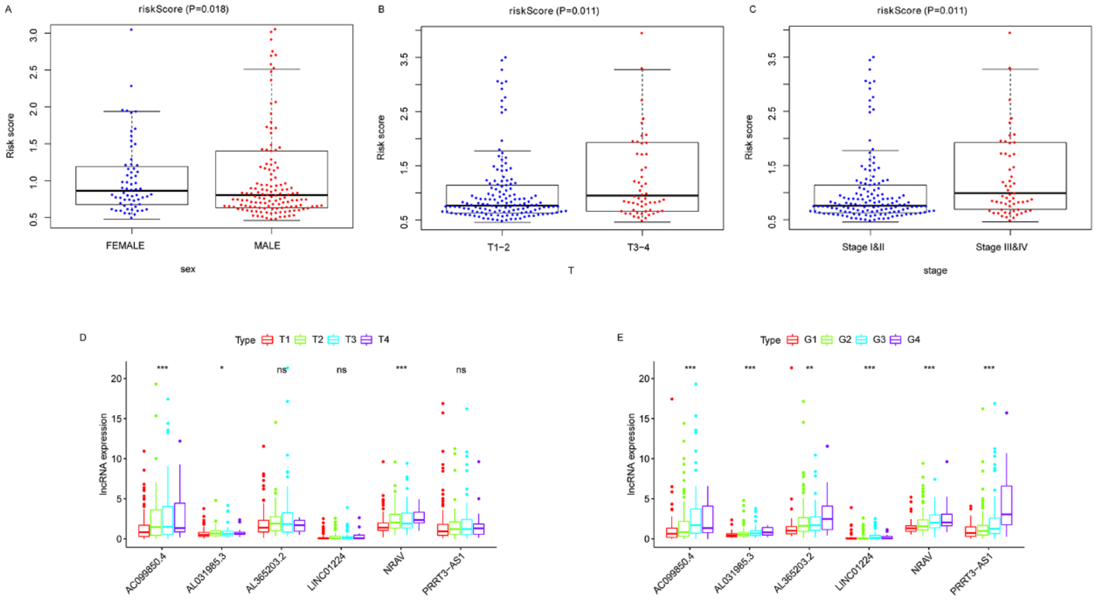

Figure S3. Association between immune-related lncRNAs, risk score and clinicopathological features in The Cancer Genome Atlas dataset. The association between risk score and (**A**) sex, (**B**) T-stage and (**C**) pathological stage. Data were analyzed using an unpaired t-test. The association between the six immune-related lncRNAs and (**D**) T-stage and (**E**) histological grade. Data were analyzed using the Kruskal-Wallis test. $^{*}P<0.05$, $^{**}P<0.01$ and $^{***}P<0.001$. lncRNA, long non-coding RNA; ns, not significant; T-stage, tumor stage; NRAV, negative regulator of antiviral response; PRRT3-AS1, PRRT3-antisense RNA1; LINC01224, long intergenic non-protein coding RNA 1224.

Table S1. Characteristics of the patients with hepatocellular carcinoma in The Cancer Genome Atlas dataset (n=371).

Characteristic	Number of patients
Age, ≤65/>65/NA, years	232/138/1
Sex, male/female	250/121
Grade, 1/2/3/4/NA	55/177/122/12/5
Stage, I/II/III/IV/NA	171/86/85/5/24
T stage, T1/T2/T3/T4/Tx/NA	181/94/80/13/1/2
N stage, N0/N1/Nx/NA	252/4/114/1
M stage, M0/M1/Mx	266/4/101

NA, not available; T, tumor; N, node; M, metastasis.

Table S2. Clinical features of six patients with hepatocellular carcinoma

Characteristic	Number of patients
Age	
≤ 65	4
>65	2
Gender	
male	4
female	2
T stage	
T1	4
T2	1
T3	1
N stage	
N0	6
N1	0
M stage	
M0	6
M1	0
Histological grade	
1	0
2	5
3	1
Stage	
I	4
II	1
IIIa	1
Pathological type	
HCC	6

Abbreviation: T, tumor; N, node; M, metastasis.
